# Application of a new PCR-RFLP panel suggests a restricted population structure for *Eimeria tenella* in UK and Irish chickens

**DOI:** 10.1016/j.vetpar.2016.09.018

**Published:** 2016-10-15

**Authors:** Elaine Pegg, Kate Doyle, Emily L. Clark, Isa D. Jatau, Fiona M. Tomley, Damer P. Blake

**Affiliations:** a Pathology and Pathogen Biology, Royal Veterinary College, Hawkshead Lane, North Mymms, AL9 7TA, UK; b Department of Parasitology and Entomology, Faculty of Veterinary Medicine, Ahmadu Bello University, Zaria, Nigeria

**Keywords:** *Eimeria tenella*, Chicken, Genetic diversity, Population structure, PCR-RFLP

## Abstract

*Eimeria* species cause coccidiosis, most notably in chickens where the global cost exceeds US$3 billion every year. Understanding variation in *Eimeria* population structure and genetic diversity contributes valuable information that can be used to minimise the impact of drug resistance and develop new, cost-effective anticoccidial vaccines. Little knowledge is currently available on the epidemiology of *Eimeria* species and strains in different regions, or under different chicken production systems. Recently, 244 *Eimeria tenella* isolates collected from countries in Africa and Asia were genotyped using a Sequenom single nucleotide polymorphism (SNP) tool, revealing significant variation in haplotype diversity and population structure, with a marked North/South regional divide. To expand studies on genetic polymorphism to larger numbers of *E. tenella* populations in other geographic regions a cheaper and more accessible technique, such as polymerase chain reaction-restriction fragment length polymorphism (PCR-RFLP), is desirable. We have converted a subset of SNP markers for use as PCR-RFLPs and re-analysed the original 244 isolates with the PCR-RFLPs to assess their utility. In addition, application of the PCR-RFLP to *E. tenella* samples collected from UK and Irish broiler chickens revealed a tightly restricted haplotype diversity. Just two of the PCR-RFLPs accounted for all of the polymorphism detected in the UK and Irish parasite populations, but analysis of the full dataset revealed different informative markers in different regions, supporting validity of the PCR-RFLP panel. The tools described here provide an accessible and cost-effective method that can be used to enhance understanding of *E. tenella* genetic diversity and population structure.

## Highlights

• *Eimeria tenella* populations differ in genetic diversity between regions.
• PCR-RFLP provides a robust tool to assess genetic diversity for *Eimeria tenella*.
• Cost-effective genotyping can support expansion of population genetics for *Eimeria.*

## Graphical abstract

**Figure:**
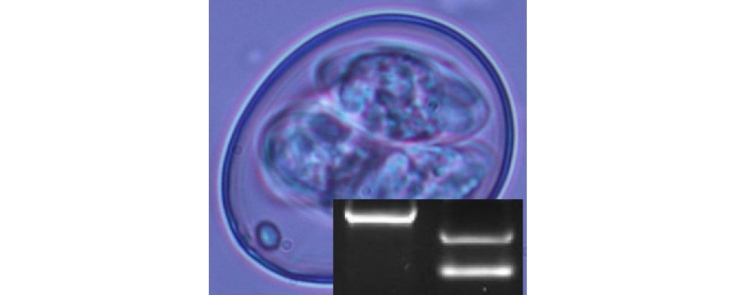

## 1 Introduction

Apicomplexan parasites can cause serious human and animal disease (e.g. Gardner et al., 2002, Shirley et al., 2005, Reid et al., 2012). Control strategies vary between genera but effective or improved vaccines are a major goal for all. Experimental recombinant or subunit vaccines have been described for many apicomplexans with varying levels of efficacy obtained under controlled laboratory or experimental animal conditions. However, commercial translation has commonly been hindered by a range of factors including naturally occurring genetic diversity in field parasites, resulting in insufficient immunological protection in the vaccinated host and selection of resistant populations (Dutta et al., 2007, Blake et al., 2015). If vaccines are to be successful and remain effective in the long term it is essential to understand the impacts that parasite genetic (antigenic) diversity and population structures have on the selection of field populations capable of vaccine escape. Studies into naturally occurring diversity have yielded notable insights for parasites such as *Plasmodium falciparum*, revealing varied levels of polymorphism with evidence of clonal, as well as panmictic structures, depending on geographic location and rates of transmission (Annan et al., 2007, Larranaga et al., 2013). Locus-specific analyses have also detected considerable diversity for *P. falciparum* vaccine candidates such as apical membrane antigen-1 and merozoite surface protein-1 (AMA1 and MSP1, (Healer et al., 2004, Simpalipan et al., 2014)). In contrast, *Toxoplasma gondii* has been defined as having a clonal population structure across much of the world apart from South America, where genetic diversity and inter-clonal interbreeding is common (Minot et al., 2012). For most other apicomplexans details on diversity are scarce; for example for *Eimeria*, the cause of the enteric disease coccidiosis, rather little is known at the molecular level. Recently, populations of *Eimeria tenella* parasites isolated from chicken facilities in Egypt, Libya, India and Nigeria were genotyped using a Sequenom MassARRAY single nucleotide polymorphism (SNP) tool, revealing notable variation in haplotype diversity and population structure with a North/South regional divide (Blake et al., 2015). *E. tenella* populations sampled below the 30th parallel north (30°N latitude) were defined by extensive haplotype diversity with no significant signatures of selection. In contrast, *E. tenella* populations sampled north of this latitude presented limited haplotype diversity with significant disequilibrium (Blake et al., 2015). More recent analysis of internal transcribed spacer (ITS) sequence diversity for *E. tenella* has confirmed this apparent variation in population structure, with notably limited diversity detected in the United States of America (USA) (Clark et al., 2016). While these data are informative, expanding research to *E. tenella* populations in other regions and laboratories requires a more accessible technique.

Genetic approaches for differentiating between parasite isolates have improved rapidly over the last 20 years. Reviewed previously (Beck et al., 2009), examples including random amplification of polymorphic DNA (RAPD), single and multi-locus sequence typing, amplified fragment length polymorphism (AFLP) and variable number tandem repeats (VNTR) analyses have all been employed with success for *Eimeria* (Fernandez et al., 2003, Schwarz et al., 2009, Blake et al., 2011, Ogedengbe et al., 2011, Lim et al., 2012). Another widely used genotyping technology is polymerase chain reaction-restriction fragment length polymorphism (PCR-RFLP). PCR-RFLP combines the sensitivity and specificity of PCR with genetic discrimination arising from substitutions, insertions and/or deletions located within recognition sites for restriction endonucleases (Rankin et al., 1996). The approach is straightforward and applicable in many laboratories given provision of a thermal cycler and equipment for gel electrophoresis. Among apicomplexan parasites PCR-RFLP has been employed most notably to genotype *T. gondii*, including examples of parasites derived from multiple host species (Su et al., 2006, Dubey et al., 2008, Burrells et al., 2016) and regions (Dubey et al., 2007a, Dubey et al., 2007b, Velmurugan et al., 2009), and underpinning some of the most fundamental population genetics studies (Su et al., 2006). The availability of more accessible and cost-effective population genetics tools will provide opportunities for complimentary, and importantly comparable, analyses of *E. tenella* from other populations.

## 2 Materials and methods

### 2.1 Reference parasites—production, processing and genomic DNA extraction

The *E. tenella* Houghton, Nippon-2, Weybridge and Wisconsin reference isolates (Shirley and Harvey, 2000, Reid et al., 2014) and a Nigerian field isolate (Jatau et al., 2016) were maintained, purified, and propagated as described previously using specific pathogen-free White Leghorn chickens (Long et al., 1976, MAFF, 1986). Genomic DNA was isolated and purified using a QIAamp DNA Stool mini kit as recommended by the manufacturer (Qiagen, Hilden, Germany), including an additional initial mechanical disruption step (Kumar et al., 2014).

### 2.2 Field sample collection—processing and genomic DNA extraction

Twenty seven field samples collected from commercial broiler farms in South/South-East England and the Republic of Ireland between 2013 and 2015 and found by previous diagnostic PCR analysis to include oocysts of *E. tenella* were available for use in this study (n = 24 and 3 respectively, diagnostic analysis not shown). Each sample was collected as described previously (Kumar et al., 2014) and consisted of pooled faecal samples collected from the floor of a poultry house, representing multiple individuals within a single flock. All farms sampled used commercial indoor broiler systems, including either Ross 308 or Cobb500 stock between four and six weeks of age (Supplementary file 1). Routine anticoccidial control was based on chemoprophylaxis on all farms sampled, with no recorded use of anticoccidial vaccination. Total oocysts (including *E. tenella*, plus any other *Eimeria* species which may have been present) were purified by saturated salt flotation, allowed to sporulate and then stored in 2.5% (w/v) potassium dichromate at +4°C. Total genomic DNA was recovered from each sample using a QIAamp mini stool DNA kit (Qiagen) as described previously (Kumar et al., 2014).

### 2.3 Sequenom marker development for PCR-RFLP

Each of the 52 *E. tenella* species-specific SNPs included in a Sequenom genotyping array (Blake et al., 2015) and found previously to yield robust profiles which supported discrimination between field isolates were assessed for development as PCR-RFLPs. The online tool NEBcutter (version 2.0, New England Biolabs; (Vincze et al., 2003)) was used to identify restriction endonuclease recognition sites within genomic DNA sequences representing 15 bp centred on each candidate SNP. All SNP-types were tested at each SNP locus. Restriction endonucleases whose recognition site was disrupted by one, but not both SNP-types were recorded for each locus, generating a series of cut/non-cut genetic markers. Where more than one restriction endonuclease was available selection was made based upon (i) activity at 37 °C, (ii) use of the generic Cut Smart buffer (New England Biolabs) and (iii) application in one or more other PCR-RFLPs to minimise complexity of the protocol. Subsequently, 1200 bp genomic sequence centred on each candidate SNP was used as template for primer design with Primer3 (Rozen and Skaletsky, 2000). Target amplicon size was set to 400–1000 bp with an optimal predicted melting temperature (Tm) centred on 60 °C. A uniform Tm was required in order to simplify application of the eventual PCR-RFLP panel. All primers were synthesised by Sigma-Aldrich (UK).

### 2.4 Polymerase chain reaction

Standard PCR amplification was completed using MyTaq DNA polymerase (Bioline). Briefly, each PCR reaction contained 1 μl template DNA (commonly containing between 10 and 50 ng), 1 μl of each of the relevant forward and reverse primers (10 μM stock) and 12.5 μl of MyTaq ×2 mastermix, made up to a final volume of 25 μl using molecular grade water (Sigma). Negative controls included molecular grade water as template. Positive controls included reference strain genomic DNA representative of each SNP type. Thermal cycler parameters were: 1× initial denaturation at 95 °C for 1 min, followed by 35× (denaturation 15 s at 95 °C, annealing 15 s at 56 °C, extension 1 min at 72 °C), followed by a final extension phase of 72 °C for 7 min. All primers used were synthesized by Sigma-Genosys and are shown in Table 1. PCR amplicons were sequenced on both strands using the same primers employed in their original amplification (GATC Biotech). Sequence assembly, annotation, and interrogation were undertaken with CLC Main Workbench v6.0.2 (CLC Bio; (CLCBioinformatics, 2015)) using BLASTn against the reference *E. tenella* Houghton genome sequence assembly to confirm identity.

### 2.5 Restriction fragment length polymorphism (RFLP)

After thermal cycling each PCR reaction underwent restriction endonuclease digestion. Briefly, 10 μl PCR product was combined with 5 U restriction enzyme (Table 1), 3 μl 10× Cut Smart buffer (New England Biolabs) and molecular biology grade water to a final volume of 30 μl, prior to incubation for 60 min at the temperature required for each enzyme (as shown in Table 1). Restriction endonucleases were purchased from New England Biolabs, using high fidelity (-HF) versions where necessary to permit use of the same Cut Smart^®^ buffer in all reactions. Subsequently, each digested PCR reaction was resolved by agarose gel electrophoresis using a 2% (w/v) UltraPure agarose gel in 1× Tris-borate-EDTA buffer (TBE; all Sigma-Aldrich), including 0.01% (v/v) SafeView nucleic acid stain (NBS Biologicals). The results of electrophoresis were visualised using a U:Genius Gel Documentation System (Syngene).

### 2.6 Genetic analysis

Following PCR-RFLP a SNP type was assigned to all eleven loci for every sample by the presence of one or two bands following electrophoresis. The presence of a single band indicated occurrence of the SNP associated with restriction endonuclease recognition site disruption. The presence of two bands indicated occurrence of the SNP associated with maintenance of the site (Table 1). Detection of both band profiles in a single sample indicated a polyclonal parasite population with genetic diversity at the target SNP (occurring between different oocysts, or the product of cross-fertilisation in hybrid oocysts). Where polyclonal patterns were detected the dominant (brightest) pattern was retained for genetic analysis. Band profiles were counted and compiled into a Microsoft Excel spreadsheet (Elliott et al., 2006). Base specificity at each SNP was identified and combined for each sample to create a SNP haplotype. Haplotypes derived here from the UK and Irish field samples were combined with the equivalent SNPs extracted from the larger Sequenom SNP panel produced prior to this study (from 244 samples collected in Egypt, India, Libya and Nigeria (Blake et al., 2015)) and converted to Phylip format using CLC Main Workbench v6.0.2.

The program DnaSP (DNA Sequence Polymorphism version 5.10.01 (Librado and Rozas, 2009)) was used to calculate the number of SNP haplotypes in the full and regional haplotype data sets. The standardised index of association (*I*_A_^S^) and associated statistical significance were calculated using LIAN v3.6 (Haubold and Hudson, 2000) as a measure of linkage disequilibrium, where values of 0 indicated no association and values significantly higher than 0 indicated association. The Monte Carlo test option was chosen with 10,000 iterations using p < 0.05 as a measure of significance. Mean genetic diversity (Het) was also calculated using LIAN. A median joining phylogenetic network was produced for the PCR-RFLP SNP haplotypes using NETWORK version 4.5.1.1 (Bandelt et al., 1999).

### 2.7 Ethics approval

This study was carried out in strict accordance with the Animals (Scientific Procedures) Act 1986, an Act of Parliament of the United Kingdom. All protocols were approved by the Royal Veterinary College Ethical Review Committee. All animal studies were approved by the United Kingdom Government Home Office.

## 3 Results

### 3.1 Identification and validation of PCR-RFLP markers for *E. tenella*

**Fig. 1:**
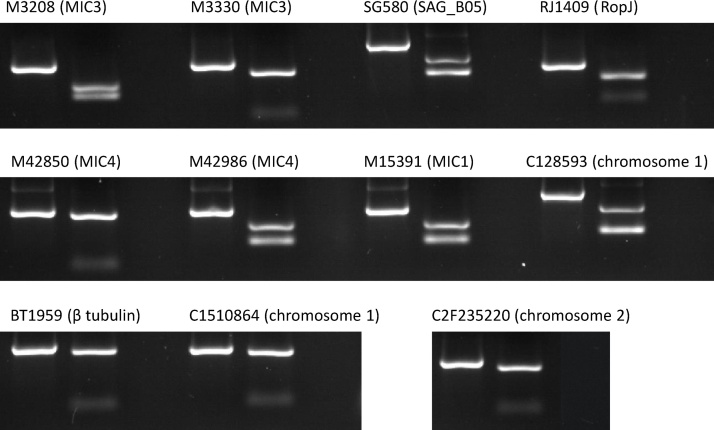
Eleven genotyping PCR-RFLPs for *Eimeria tenella*, showing uncut/cut genotypes and negative control for each. The marker name and locus (in parentheses) is shown above each set.

Fifty-two SNPs developed previously for use in a Sequenom MassARRAY SNP typing assay for *E. tenella* were assessed here for development as PCR-RFLPs. Eleven SNPs were prioritised following *in silico* identification of an appropriate restriction endonuclease and accompanying locus specific primer pair (Table 1). Direct sequencing of each PCR amplicon derived using genomic DNA extracted from the *E. tenella* Houghton, Nippon-2, Weybridge or Wisconsin reference strains, and a Nigerian field isolate, indicated amplification of a single, correct target for each PCR-RFLP when compared with the reference Houghton strain genome sequence assembly and confirmed SNP identity. Restriction endonuclease digestion confirmed the uncut/cut electrophoresis profiles for each PCR-RFLP (Fig. 1, Table 2).

### 3.2 PCR-RFLP application to UK and Irish field isolates

**Fig. 2:**
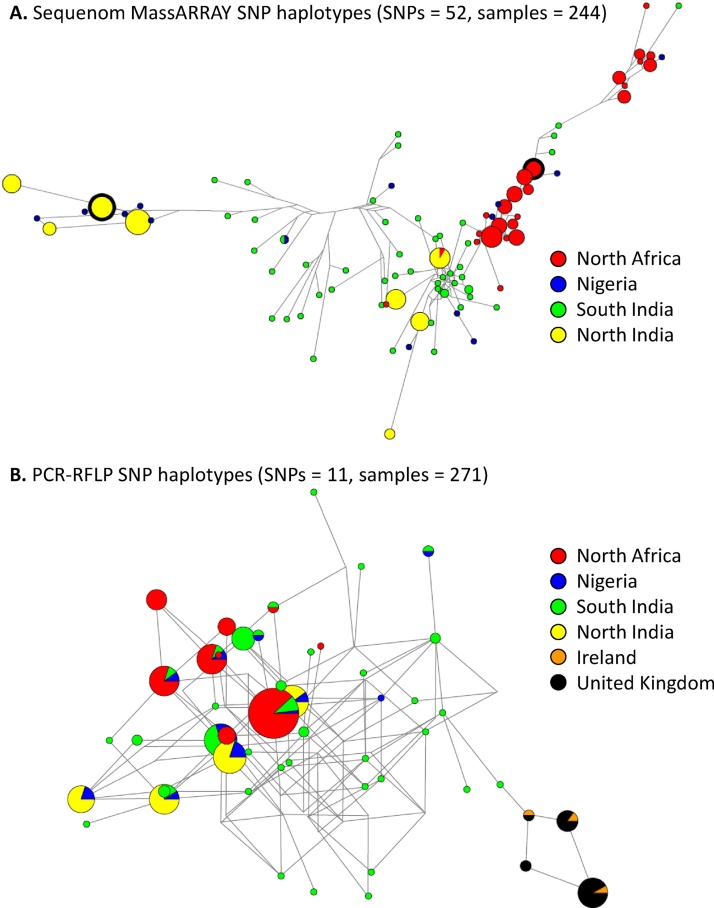
Median-joining phylogenetic NETWORKs illustrating *Eimeria tenella* SNP haplotype diversity assessed using (A) 52 SNP Sequenom MassARRAY or (B) 11 SNP PCR-RFLP genotyping protocols. Both illustrate a regional bias in haplotype occurrence with a small number of highly represented haplotypes detected in North Africa and northern India, and a much larger number of mostly unique haplotypes in southern India and Nigeria. Haplotypes from the UK and Ireland were only available using PCR-RFLP and appear distinct from the other regions. Note the incomplete NETWORK resolution achieved using the smaller number of PCR-RFLPs, indicated by multiple cycles or cubes. Node size indicates the frequency of haplotype occurrence. Panel A reproduced from (Blake et al., 2015).

Twenty seven samples collected from UK and Irish broiler farms (n = 24 and 3 respectively) and found previously by PCR to contain *E. tenella* were genotyped using the panel of 11 PCR-RFLPs. All samples were successfully genotyped at all 11 loci with no evidence of polyclonal populations (Supplementary file 1). Analysis of the PCR-RFLP profiles revealed four distinct haplotypes in the UK and Irish sample set, three of which were shared between the UK and Ireland (Fig. 2). Mean genetic diversity was low, in agreement with the limited number of haplotypes detected, although the *I*_A_^S^ provided significant evidence of disequilibrium (Table 3).

### 3.3 Comparison of genetic analyses using PCR-RFLP or sequenom SNP markers

Previous analysis of *E. tenella* genetic diversity has been based on a panel of 52 SNP markers, assessed using Sequenom MassARRAY genotyping (Blake et al., 2015). In recognition of the difficulty many laboratories may experience employing the same technology we have compared results achieved previously using the full Sequenom panel with the subset of SNPs included in the 11 PCR-RFLPs from the same samples. The variation in *E. tenella* haplotype diversity and population structure described previously for North Africa and northern India, compared to Nigeria and southern India remained distinct, indicated by the total number of haplotypes, mean genetic diversity and *I*_A_^S^ (Table 3). Populations exhibiting statistically significant disequilibrium were confirmed, despite an ∼five-fold reduction in markers. However, the regional specificity was less obvious, with greater haplotype overlap between regions. NETWORK analysis of the PCR-RFLP SNP subset of Sequenom markers illustrated these findings (compare Fig. 2A and B). The previous full Sequenom panel revealed non-overlapping *E. tenella* populations defined by the frequent occurrence of a small number of haplotypes in North Africa and northern India, complemented by many diverse, often unique haplotypes from Nigeria and southern India (Blake et al., 2015 and Fig. 2A). Analysis of the PCR RFLP SNPs presented a similar overview of haplotype occurrence but the NETWORK remained unresolved, presenting multiple high-dimensional cubes and cycles (Fig. 2B) which persisted even when using Maximum Parsimony. Only removal of the unique haplotypes permitted successful NETWORK construction at the expense of a complete portrayal of diversity. *E. tenella* PCR-RFLP SNP profiles from the UK and Ireland were notably distinct from those described in Africa and India (Fig. 2B).

### 3.4 Identification of optimal PCR-RFLP assays by region

Comparison of PCR-RFLP SNP profiles between regions revealed considerable variation in discriminatory power. Every PCR-RFLP was found to be polymorphic in at least one region, although no single SNP was able to differentiate between genotypes in every region sampled (Table 4). Only two (M3208 and C2F235220) provided discrimination between genotypes in the UK and Ireland. Markers considered most discriminatory within each region (assessed as the closest to 50% representation of each SNP-type) were less discriminatory elsewhere.

## 4 Discussion

Understanding variation in population structure and genetic diversity for pathogens such as *Eimeria* is important. As we seek to combat drug resistance and develop new, cost-effective anticoccidial vaccines for use with chickens, defining the epidemiology of parasite occurrence and disease in different regions and under different production systems has become essential. The apparently obligate requirement for sexual reproduction in eimerian life cycles (Walker et al., 2013), together with the vast number of immunologically naïve definitive hosts (chickens) produced every year, offers the potential for co-infection, resegregation/recombination and the generation of diversity among *Eimeria* strains, although the extent to which this occurs remains largely unknown. The development of a PCR-RFLP panel for the related coccidian *T. gondii* provides an excellent example of how having accessible population genetics tools provides opportunities for comparable analyses of parasites from around the world. There are more than 170 peer reviewed manuscripts available in PubMed under the keywords ‘*Toxoplasma’* and ‘PCR-RFLP’ (accessed 12th July; 2016); including surveillance of multiple host species in many different regions (e.g. Su et al., 2006, Dubey et al., 2007a, Dubey et al., 2007b, Dubey et al., 2008, Velmurugan et al., 2009, Burrells et al., 2016). Development of PCR-RFLP for *Eimeria*; starting with *E. tenella*; will allow expansion of genetics studies with these parasites. Sequencing the PCR-RFLP loci from a panel of reference isolates confirmed the presence of the target SNPs; and provides a robust platform for application of the assay to field isolates.

Recent comparison of *E. tenella* populations in Africa and India found that samples collected in the more northern regions (Egypt, Libya and northern India) displayed less variation in genome-wide genetic diversity and population structure than those from southern regions (Nigeria and southern India) (Blake et al., 2015). Extension of this survey to include *E. tenella* populations collected from broilers reared in the UK and Ireland now reveals restricted genetic diversity with significant evidence of linkage disequilibrium comparable to the more northern regions sampled previously. It is important to note that just three samples from Ireland were characterised in this study, indicating that additional uncaptured diversity may occur. However, all three Irish haplotypes were also detected in the UK suggesting that *E. tenella* from Ireland and the UK represent one genetic population. Further sampling will be required to confirm this view. The absence of polyclonal *E. tenella* populations in this study may be a consequence of low levels of genetic diversity within the sampled regions, although this can also not be confirmed here. The reasons for reduced diversity among northern *E. tenella* such as those sampled in the UK and Ireland, compared with more southern populations, remain unclear. It is possible that regional variations may exist in biosecurity and the application of anticoccidial control measures, although direct evidence of this is limited at present. Chemoprophylaxis remains the dominant form of control in all regions tested with a variety of ionophore and chemical products (Blake and Tomley, 2014), although it has been noted that proportional inclusion of the active ingredients can be highly variable (de Gussem et al., 2007, Alcala-Canto et al., 2014). Anticoccidial vaccination using live *Eimeria* parasites is widespread in European breeder and layer stock, but remains uncommon in the majority broiler sector. Vaccines are also used in Egypt, Nigeria and India, but with a lower overall uptake than chemoprophylaxis and with a similar bias against broilers. Regional differences in climate may also be important, with the humid tropical climate in the southern regions sampled possibly favouring oocyst sporulation and persistence (Deichmann and Eklundh, 1991, Awais et al., 2012), and thus encouraging co-infection. The expansion of surveillance to other northern and southern regions is required to test these hypotheses, although it is noteworthy that the UK and US reference strains tested here differed at just two PCR-RFLP SNPs, both of which were found to be variant within UK and Irish broilers. Interestingly, comparison with the UK reference strains genotyped during validation highlighted notably different profiles with three more SNPs found to be polymorphic than in the recent field samples (compare Table 2, Table 4, also Supplementary file 1). The *E. tenella* Houghton and Weybridge strains were both isolated more than 60 years ago (Chapman and Shirley, 2003), providing considerable scope for mutation and genome evolution.

Comparison of PCR-RFLP SNP profiles highlighted varied discriminatory power in different regions. In routine or pilot studies knowledge of the most informative SNPs offers the opportunity to screen using a streamlined, region-specific panel to reduce reagent costs and operator time. For genetic analysis additional markers are likely to be required. Comparison of the 52 SNP Sequenom and 11 SNP PCR-RFLP panels revealed equivalent haplotype numbers, mean genetic diversity and significance of association within each population, albeit with less focus on regional specificity. While NETWORK analysis was not fully resolved, the results achieved here support the validity of the full PCR-RFLP panel. Nonetheless, confirmation of the limited diversity discovered among UK and Irish *E. tenella* would benefit from adoption of additional markers given that just two of the eleven developed here proved informative.

## 5 Conclusion

As next generation sequencing technologies develop and become more cost-effective it is anticipated that metagenomics approaches focused on complex field samples will eventually become available for *Eimeria*. However, such tools remain distant for many veterinary pathogens. For now PCR-RFLP can begin to fill an important knowledge gap, exploring aspects of genetic diversity and population structure with direct relevance to anticoccidial control. Application of the technology to *Eimeria* from different countries and production systems can provide an important dataset whose value will be enhanced given the capture of key variables such as system type, chicken breed and age, date of sampling and farm choice of anticoccidial control.

## Conflict of interest statement

The authors declare that they have no competing interests.

## Figures and Tables

**Table 1:** Polymerase chain reaction-restriction fragment length (PCR-RFLP) markers for *Eimeria tenella*.

Marker	Target locus	Genome contig^a^	Primers (5′ − 3′)	Amplicon (bp)	Restriction Enzyme (reaction temperature)	Target site [SNP] Underlined = cuts	Digest fragments (bp)
M3208	Microneme protein 3 (MIC3)	HG675067	F: CACTTGAGTCCACTGCTCCA R: TCATTGACAGCGACAAAAGC	504	*Dde*I (37 °C)	[C/T]TNAG	293/211
M3330	Microneme protein 3 (MIC3)	HG675067	As M3208	As M3208	*Mbo*II (37 °C)	[G/A]AAGA	416/88
SG580	Surface antigen SAG_B05	HG673747	F: TGAACCAGTTCAACGCAGAC R: CCTGATGGCCACTGAGAAAT	935	*Bts*CI (50 °C)	GGAT[G/A]	558/377
RJ1409	Rhoptry protein J (RopJ)	HG677946	F: GACTTGGTACGTTGGCCACT R: TTCCAATGTCCTTGCCTTTC	454	*Eco*RV-HF (37 °C)	GA[T/A]ATC	315/139
M42850	Microneme protein 4 (MIC4)	HG675525	F: GACATCGACGAATGTGCAAG R: GTACGAACCCGCAGTGTTTT	612	*Hind*III-HF (37 °C)	A[A/G]GCTT	519/93
M42986	Microneme protein 4 (MIC4)	HG675525	As M42850	As M42850	*Fau*I (55 °C)	CCCG[C/G]	383/229
M15391	Microneme protein 1 (MIC1)	HG673835	F: CGAACAGGACAAATGGTGTG R: GTTGGGAGTCTGCACAGTGA	617	*Eco*RI-HF (37 °C)	GAATT[C/A]	386/231
BT1959	β-tubulin	HG673764	F: ATGCTTCCCCCTGAATCTTT R: TATTCTTCGCGGACTTTGCT	642	*Bsr*BI (37 °C)	CCGCT[C/G]	598/44
C128593	Chromosome 1, feature-poor region 1 (C1P1)	HG675753	F: AATTAAAAGAAGGCGCAGCA R: CATACAAGCACCCAATGACG	889	*Bts*IMutI (55 °C)	CA[G/C]TG	592/297
C1510864	Chromosome 1, feature-rich region 1 (C1R1)	HG675718	F: CTGCAGTTTGCTGCTTCTTG R: GAGGCCCCATTATAGCTTCC	599	*Bts*IMutI (55 °C)	CA[G/A]TG	544/55
C2F235220	Chromosome 2, feature-poor region 1 (C2P1)	HG673763	F: GCACCAGCTGAACGTTTGTA R: GAGAGCACGAACAACAACGA	520	*Bts*IMutI (55 °C)	CA[G/A]TG	444/76

a Eimeria tenella Houghton strain genome, version 2013-11-05 (accessed through ToxoDB Gajria et al., 2008).

**Table 2:** PCR-RFLP profiles defining five reference and field *Eimeria tenella* isolates. I = PCR-RFLP cut profile, II = PCR-RFLP uncut profile.

Origin:	UK	UK	USA	Nigeria	Japan
Marker	Houghton (H)	Weybridge (Wey)	Wisconsin (Wis)	Nigeria-1 (Nig1)	Nippon-2 (Nt2)
M3208	I	I	II	I	I
M3330	I	I	I	II	I
SG580	I	I	I	I	II
RJ1409	II	II	II	II	I
M42850	II	II	II	I	I
M42986	I	I	I	II	II
M15391	I	I	I	I	II
BT1959	I	I	I	II	I
C128593	II	II	II	II	I
C1510864	II	II	II	I	I
C2F235220	II	II	I	I	I

**Table 3:** Summary of *Eimeria tenella* genome-wide genetic data calculated using previous Sequenom MassARRAY genotyping (including 52 SNPs per sample; top figure in each cell as reported previously (Blake et al., 2015)) or PCR-RFLP (11 SNPs per sample; bottom *italicised figure* in each cell).

			SNP haplotypes				
Region	Assay	N	Total	Region specific	Het (+/−)	I_A_^S^	*p*
All (no UK)	Sequenom	244	93	–	0.361 (0.019)	0.124	<0.001
	*PCR-RFLP*	*244*	*48*	*–*	*0.351 (0.046)*	*0.164*	*<0.001*

All (Inc. UK)	Sequenom	nd	nd	nd	nd	nd	nd
	*PCR-RFLP*	*271*	*52*	*–*	*0.388 (0.031)*	*0.140*	*<0.001*

N. India	Sequenom	86	8	7	0.333 (0.025)	0.115	<0.001
	*PCR-RFLP*	*86*	*8*	*2*	*0.276 (0.069)*	*0.203*	*<0.001*

S. India	Sequenom	53	50	49	0.340 (0.021)	0.009	ns
	*PCR-RFLP*	*53*	*36*	*27*	*0.314 (0.040)*	*0.022*	*ns*

Egypt	Sequenom	40	21	13	0.192 (0.032)	0.058	<0.001
	*PCR-RFLP*	*40*	*5*	*1*	*0.135 (0.070)*	*0.072*	*<0.001*

Libya	Sequenom	51	11	3	0.161 (0.025)	0.053	<0.001
	*PCR-RFLP*	*51*	*8*	*3*	*0.138 (0.057)*	*0.117*	*<0.001*

Egypt + Libya	Sequenom	91	25	24	0.179 (0.027)	0.053	<0.001
	*PCR-RFLP*	91	9	5	0.139 (0.061)	*0.084*	*<0.001*

Nigeria	Sequenom	14	14	13	0.400 (0.0224)	0.001	ns
	*PCR-RFLP*	*14*	*11*	*1*	*0.3916 (0.052)*	*0.013*	*ns*

UK + Ireland	Sequenom	nd	nd	nd	nd	nd	nd
	*PCR-RFLP*	*27*	*4*	*4*	*0.068 (0.048)*	*0.0021*	*<0.0001*

N = the number of samples tested. The number of SNP haplotypes was calculated using DnaSP v5.10.01. Het = mean genetic diversity and I_A_^S^ = standardised index of association, with statistical significance indicated as p, calculated using LIAN v3.6. nd = not done, ns = not statistically significant.

**Table 4:** Summary of PCR-RFLP genotypes from field samples found to contain *Eimeria tenella*. The dominant SNP type detected is shown for each region, together with the percentage of samples defined by that SNP type.

	Dominant SNP type (% samples)				
Marker	North India (n = 86)	South India (n = 53)	North Africa (n = 91)	Nigeria (n = 14)	UK + Ireland (n = 27)
M3208	C (58.1)	T (75.5)	T (98.9)	C/T (50.0)^a^	T (85.2)
M3330	G (68.6)	G (60.3)^a^	G (92.3)	G (78.6)	G (100.0)
SG580	A (61.6)	G (64.1)	G (98.9)	A/G (50.0)^a^	A (100.0)
RJ1409	C (100.0)	C (75.5)	C (100.0)	C (92.9)	T (100.0)
M42850	G (59.3)	G (88.7)	G (100.0)	G (71.4)	G (100.0)
M42986	C (73.3)	G (92.4)	G (100.0)	C/G (50.0)^a^	G (100.0)
M15391	A (86.0)	C (73.6)	C (100.0)	A (71.4)	A (100.0)
BT1959	G (100.0)	G (96.2)	G (100.0)	G (100.0)	C (100.0)
C128593	C (52.3)^a^	G (83.0)	G (64.8)	G (64.3)	C (100.0)
C1510864	A (100.0)	A (75.5)	G (56.0)^a^	A (78.6)	G (100.0)
C2F235220	G (100.0)	G (84.9)	A (74.7)	G (71.4)	A (63.0)^a^

a Most discriminatory marker within each region (column).

## Appendix A Supplementary data

Supplementary Table S1 Detailed PCR-RFLP results and accompanying sample data. Collection details for each sample analysed in these studies and the PCR-RFLP profiles derived.

## References

[bib0005] Alcala-Canto Y., Ramos-Martinez E., Tapia-Perez G., Gutierrez L., Sumano H. (2014). Pharmacodynamic evaluation of a reference and a generic toltrazuril preparation in broilers experimentally infected with *Eimeria tenella* or *E. acervulina*. Br. Poult. Sci..

[bib0010] Annan Z., Durand P., Ayala F.J., Arnathau C., Awono-Ambene P., Simard F., Razakandrainibe F.G., Koella J.C., Fontenille D., Renaud F. (2007). Population genetic structure of *Plasmodium falciparum* in the two main African vectors, Anopheles gambiae and Anopheles funestus. Proc. Natl. Acad. Sci. U. S. A..

[bib0015] Awais M.M., Akhtar M., Iqbal Z., Muhammad F., Anwar M.I. (2012). Seasonal prevalence of coccidiosis in industrial broiler chickens in Faisalabad Punjab, Pakistan. Trop. Anim. Health Prod..

[bib0020] Bandelt H.J., Forster P., Rohl A. (1999). Median-joining networks for inferring intraspecific phylogenies. Mol. Biol. Evol..

[bib0025] Beck H.P., Blake D., Darde M.L., Felger I., Pedraza-Diaz S., Regidor-Cerrillo J., Gomez-Bautista M., Ortega-Mora L.M., Putignani L., Shiels B., Tait A., Weir W. (2009). Molecular approaches to diversity of populations of apicomplexan parasites. Int. J. Parasitol..

[bib0030] Blake D.P., Tomley F.M. (2014). Securing poultry production from the ever-present *Eimeria* challenge. Trends Parasitol..

[bib0035] Blake D.P., Billington K.J., Copestake S.L., Oakes R.D., Quail M.A., Wan K.L., Shirley M.W., Smith A.L. (2011). Genetic mapping identifies novel highly protective antigens for an apicomplexan parasite. PLoS Pathog..

[bib0040] Blake D., Clark E., Macdonald S., Thenmozhi V., Kundu K., Garg R., Jatau I., Ayoade S., Kawahara F., Moftah A., Reid A., Adebambo A., Álvarez-Zapata R., Srinivasa Rao A., Thangaraj K., Banerjee P., Dhinakar-Raj G., Raman M., Tomley F. (2015). Population, genetic and antigenic diversity of the apicomplexan *Eimeria tenella* and their relevance to vaccine development. Proc. Natl. Acad. Sci. U. S. A..

[bib0045] Burrells A., Opsteegh M., Pollock K.G., Alexander C.L., Chatterton J., Evans R., Walker R., McKenzie C.A., Hill D., Innes E.A., Katzer F. (2016). The prevalence and genotypic analysis of *Toxoplasma gondii* from individuals in Scotland, 2006–2012. Parasit. Vectors.

[bib0050] CLCBioinformatics, 2015. CLC Main Workbench Version 5.

[bib0055] Chapman H., Shirley M. (2003). The Houghton strain of *Eimeria tenella*: a review of the type strain selected for genome sequencing. Avian Pathol..

[bib0060] Clark E.L., Macdonald S.E., Thenmozhi V., Kundu K., Garg R., Kumar S., Ayoade S., Fornace K.M., Jatau I.D., Moftah A., Nolan M.J., Sudhakar N.R., Adebambo A.O., Lawal I.A., Alvarez Zapata R., Awuni J.A., Chapman H.D., Karimuribo E., Mugasa C.M., Namangala B., Rushton J., Suo X., Thangaraj K., Srinivasa Rao A.S., Tewari A.K., Banerjee P.S., Dhinakar Raj G., Raman M., Tomley F.M., Blake D.P. (2016). Cryptic *Eimeria* genotypes are common across the southern but not northern hemisphere. Int. J. Parasitol..

[bib0065] Deichmann U., Eklundh L. (1991). Global Digital Data Sets for Land Degradation Studies: A GIS Approach.

[bib0070] Dubey J.P., Cortes-Vecino J.A., Vargas-Duarte J.J., Sundar N., Velmurugan G.V., Bandini L.M., Polo L.J., Zambrano L., Mora L.E., Kwok O.C., Smith T., Su C. (2007). Prevalence of *Toxoplasma gondii* in dogs from Colombia: south America and genetic characterization of T. gondii isolates. Vet. Parasitol..

[bib0075] Dubey J.P., Sundar N., Nolden C.A., Samuel M.D., Velmurugan G.V., Bandini L.A., Kwok O.C., Bodenstein B., Su C. (2007). Characterization of *Toxoplasma gondii* from raccoons (*Procyon lotor*), coyotes (*Canis latrans*), and striped skunks (*Mephitis mephitis*) in Wisconsin identified several atypical genotypes. J. Parasitol..

[bib0080] Dubey J.P., Velmurugan G.V., Chockalingam A., Pena H.F., de Oliveira L.N., Leifer C.A., Gennari S.M., Bahia Oliveira L.M., Su C. (2008). Genetic diversity of *Toxoplasma gondii* isolates from chickens from Brazil. Vet. Parasitol..

[bib0085] Dutta S., Lee S.Y., Batchelor A.H., Lanar D.E. (2007). Structural basis of antigenic escape of a malaria vaccine candidate. Proc. Natl. Acad. Sci. U. S. A..

[bib0090] Elliott A.C., Hynan L.S., Reisch J.S., Smith J.P. (2006). Preparing data for analysis using microsoft Excel. J. Investig. Med..

[bib0095] Fernandez S., Costa A.C., Katsuyama A.M., Madeira A.M., Gruber A. (2003). A survey of the inter- and intraspecific RAPD markers of *Eimeria* spp. of the domestic fowl and the development of reliable diagnostic tools. Parasitol. Res..

[bib0100] Gajria B., Bahl A., Brestelli J., Dommer J., Fischer S., Gao X., Heiges M., Iodice J., Kissinger J.C., Mackey A.J., Pinney D.F., Roos D.S., Stoeckert C.J., Wang H., Brunk B.P. (2008). ToxoDB: an integrated *Toxoplasma gondii* database resource. Nucleic Acids Res..

[bib0105] Gardner M.J., Hall N., Fung E., White O., Berriman M., Hyman R.W., Carlton J.M., Pain A., Nelson K.E., Bowman S., Paulsen I.T., James K., Eisen J.A., Rutherford K., Salzberg S.L., Craig A., Kyes S., Chan M.S., Nene V., Shallom S.J., Suh B., Peterson J., Angiuoli S., Pertea M., Allen J., Selengut J., Haft D., Mather M.W., Vaidya A.B., Martin D.M., Fairlamb A.H., Fraunholz M.J., Roos D.S., Ralph S.A., McFadden G.I., Cummings L.M., Subramanian G.M., Mungall C., Venter J.C., Carucci D.J., Hoffman S.L., Newbold C., Davis R.W., Fraser C.M., Barrell B. (2002). Genome sequence of the human malaria parasite *Plasmodium falciparum*. Nature.

[bib0110] Haubold B., Hudson R.R. (2000). LIAN 3.0: detecting linkage disequilibrium in multilocus data. Linkage analysis. Bioinformatics.

[bib0115] Healer J., Murphy V., Hodder A., Masciantonio R., Gemmill A., Anders R., Cowman A., Batchelor A. (2004). Allelic polymorphisms in apical membrane antigen-1 are responsible for evasion of antibody-mediated inhibition in *Plasmodium falciparum*. Mol. Microbiol..

[bib0120] Jatau I.D., Lawal I.A., Kwaga J.K., Tomley F.M., Blake D.P., Nok A.J. (2016). Three operational taxonomic units of *Eimeria* are common in Nigerian chickens and may undermine effective molecular diagnosis of coccidiosis. BMC Vet. Res..

[bib0125] Kumar S., Garg R., Moftah A., Clark E.L., Macdonald S.E., Chaudhry A.S., Sparagano O., Banerjee P.S., Kundu K., Tomley F.M., Blake D.P. (2014). An optimised protocol for molecular identification of *Eimeria* from chickens. Vet. Parasitol..

[bib0130] Larranaga N., Mejia R.E., Hormaza J.I., Montoya A., Soto A., Fontecha G.A. (2013). Genetic structure of *Plasmodium falciparum* populations across the Honduras-Nicaragua border. Malaria J..

[bib0135] Librado P., Rozas J. (2009). DnaSP v5: a software for comprehensive analysis of DNA polymorphism data. Bioinformatics.

[bib0140] Lim L.S., Tay Y.L., Alias H., Wan K.L., Dear P.H. (2012). Insights into the genome structure and copy-number variation of *Eimeria tenella*. BMC Genomics.

[bib0145] Long P., Joyner L., Millard B., Norton C. (1976). A guide to laboratory techniques used in the study and diagnosis of avian coccidiosis. Folia Vet. Lat..

[bib0150] MAFF, (1986). Manual of Veterinary Parasitological Laboratory Techniques.

[bib0155] Minot S., Melo M.B., Li F., Lu D., Niedelman W., Levine S.S., Saeij J.P. (2012). Admixture and recombination among *Toxoplasma gondii* lineages explain global genome diversity. Proc. Natl. Acad. Sci. U. S. A..

[bib0160] Ogedengbe J.D., Hanner R.H., Barta J.R. (2011). DNA barcoding identifies *Eimeria* species and contributes to the phylogenetics of coccidian parasites (Eimeriorina, Apicomplexa, Alveolata). Int. J. Parasitol..

[bib0165] Rankin D.R., Narveson S.D., Birkby W.H., Lai J. (1996). Restriction fragment length polymorphism (RFLP) analysis on DNA from human compact bone. J. Forensic Sci..

[bib0170] Reid A.J., Vermont S.J., Cotton J.A., Harris D., Hill-Cawthorne G.A., Konen-Waisman S., Latham S.M., Mourier T., Norton R., Quail M.A., Sanders M., Shanmugam D., Sohal A., Wasmuth J.D., Brunk B., Grigg M.E., Howard J.C., Parkinson J., Roos D.S., Trees A.J., Berriman M., Pain A., Wastling J.M. (2012). Comparative genomics of the apicomplexan parasites *Toxoplasma gondii* and *Neospora caninum*: Coccidia differing in host range and transmission strategy. PLoS Pathog..

[bib0175] Reid A., Blake D., Ansari H., Billington K., Browne H., Dunn M., Hung S., Kawahara F., Miranda-Saavedra D., Malas T., Mourier T., Nagra H., Nair M., Otto T., Rawlings N., Rivailler P., Sanchez-Flores A., Sanders M., Subramaniam C., Tay Y.-L., Wu X., Dear P., Doerig C., Gruber A., Ivens A., Parkinson J., Shirley M., Wan K.-L., Berriman M., Tomley F., Pain A. (2014). Genomic analysis of the causative agents of coccidiosis in domestic chickens. Genome Res..

[bib0180] Rozen S., Skaletsky H. (2000). Primer3 on the WWW for general users and for biologist programmers. Methods Mol. Biol..

[bib0185] Schwarz R.S., Jenkins M.C., Klopp S., Miska K.B. (2009). Genomic analysis of *Eimeria* spp. populations in relation to performance levels of broiler chicken farms in Arkansas and North Carolina. J. Parasitol..

[bib0190] Shirley M.W., Harvey D.A. (2000). A genetic linkage map of the apicomplexan protozoan parasite *Eimeria tenella*. Genome Res..

[bib0195] Shirley M.W., Smith A.L., Tomley F.M. (2005). The biology of avian *Eimeria* with an emphasis on their control by vaccination. Adv. Parasitol..

[bib0200] Simpalipan P., Pattaradilokrat S., Siripoon N., Seugorn A., Kaewthamasorn M., Butcher R.D., Harnyuttanakorn P. (2014). Diversity and population structure of *Plasmodium falciparum* in Thailand based on the spatial and temporal haplotype patterns of the C-terminal 19-kDa domain of merozoite surface protein-1. Malaria J..

[bib0205] Su C., Zhang X., Dubey J.P. (2006). Genotyping of *Toxoplasma gondii* by multilocus PCR-RFLP markers: a high resolution and simple method for identification of parasites. Int. J. Parasitol..

[bib0210] Velmurugan G.V., Su C., Dubey J.P. (2009). Isolate designation and characterization of *Toxoplasma gondii* isolates from pigs in the United States. J. Parasitol..

[bib0215] Vincze T., Posfai J., Roberts R.J. (2003). NEBcutter: a program to cleave DNA with restriction enzymes. Nucleic Acids Res..

[bib0220] Walker R.A., Ferguson D.J., Miller C.M., Smith N.C. (2013). Sex and *Eimeria*: a molecular perspective. Parasitology.

[bib0225] de Gussem M., Vancraeynest D., van der Meeren P., Marien M. (2007). Differences between generic and brand specific approved (BSA) anticoccidials. In 16th European Symposium on Poultry Nutrition.

